# Situating stigma: Accounting for deviancy, difference and categorial relations

**DOI:** 10.1111/jep.13749

**Published:** 2022-08-25

**Authors:** Robin James Smith, Paul Atkinson, Rhiannon Evans

**Affiliations:** ^1^ School of Social Sciences Cardiff University Cardiff UK

**Keywords:** care‐experienced children, categorisation, practical reasoning, reasoning, social theory, stigma

## Abstract

This article returns to Goffman's early formulations of ‘stigma’ in outlining a critique of contemporary social scientific uses and abuses of the concept. We argue that whilst Goffman's discussion of stigma is not without its troubles, it has mostly been approached in a manner that treats the concept outside of an appreciation of stigma as a phenomenon of interaction order. More specifically, we discuss and demonstrate how stigma serves an analytic gloss for social relations observable in social settings and in accounts of difference, deviance and degradation. We analyse both social scientific and lay uses of the stigma concept in relation to care‐experienced young children and self‐harm to demonstrate the shared categorisational practices and logics that are often obscured through theoretical treatments of stigma. The recommendation is, then, that an attention to ‘stigma’ in care settings must begin with the conditions in and from which stigma might come to feature as a sense‐making device for all parties.

## 1 INTRODUCTION

The concept of stigma has, since the seminal writings of Goffman,^1^ endured a varied career (see Muller^2^). It is a career recently undergoing a renaissance of sorts which finds stigma enroled as an explanatory concept for an array of social relations and experiences. It is, however, increasingly applied without the kind of conceptual accuracy that Goffman^3^ stressed ‘students of society’ should strive for. Critiques, readings and revisions of Goffman's work in relation to stigma are routinely produced without regard for the project that Goffman was pursuing; the delineation of a situational sociology that took seriously the interaction order, in its own right.^4^ Anthony Giddens^5^ once wrote that he was setting out to rescue Goffman from his fans. In this article, we are not quite setting out to rescue Goffman from his critics, but we do intend to make serious use of Goffman's formulations of stigma as a matter of social *relations*. In that sense, we contribute to arguments for understanding stigma as a thoroughly interactional and situational matter.^6^ In contrast to dominant contemporary treatments, stigma ought to be treated as *belonging* to interactions and settings, not a discrete ‘thing’ that is attached to individuals. Stigma is a product of interaction or, to put things another way, an *accomplishment*.^7^ In doing so, we do not analyse clinical interactions themselves, but, instead, describe some of the work that the stigma concept is put to—as an explanatory device and theoretical gloss in formal social science analyses and by lay members—in making sense of difficult, shaming or degrading experiences.

In attending to how stigma is talked about and ‘used’ as an explanatory device, we pay particular attention here to membership categorization practices.^8^, ^9^, ^10^, ^11^ Membership categorization practices are, among other things, the methods though which members of a given scene come to categorize—and thus make sense of—fellow members. Members' selections of relevant categories are thus foundational for social organization. As such, it seems to us that such practices must be fundamental to the interactional production of stigma. The import of the approach is that, rather than assuming that certain categories are relevant and ‘bound’ to stigma at all points, we argue for an attention to the detail of interaction and people's *actual* orientations to categories which, in turn, reveals the grounds upon which stigma is produced.

In what follows, we describe some of the ‘categorial logics’ present in both social scientific and lay uses of the stigma concept. Our first set of examples are drawn from social science publications. Here, we show something of the heavy lifting that the stigma concept is made to do. Our second set of examples are drawn from interviews with carers of young people, and their accounts of those young people's treatment, and exclusion, by others. Here, we show how the stigma concept is deployed to make sense of the troubles of those with experience of being in care. In drawing from both formal social scientific and lay uses of the concept, we demonstrate a family resemblance in the work the concept is put to; work that is grounded in the same categorial logics.

The pay‐off of our discussion is recognizing that troubles of shame, degradation, exclusion and so on, deserve careful attention which, perhaps ironically, can be obscured by using stigma as a catch‐all gloss. We begin with a different order of trouble, and a discussion of the career of the concept of stigma in mainstream social scientific usage.

### 1.1 Stigma trouble: recovering and topicalising Goffman's formulations

Over the course of its career—a career increasingly independent of Erving Goffman's original formulations—the concept of ‘stigma’ has become widely diffused. The term itself, of course, predates Goffman's discussion. It is a term ‘out there’ in society which, through its very familiarity, causes issues of clarity and consistency when applied in a social scientific context. It is a ‘natural language’ concept *par excellence*. In his use, Goffman^1^ makes a careful delineation between the common usage of the term and the work he puts the concept to in his own writing, emphasizing that ‘it should be seen that a language of relationships, not attributes, is really needed’ other words, we need to understand that any given attribute is not necessarily stigmatic in own right but becomes so in and through situated social relations.

Despite the apparent clarity of Goffman's opening remarks, the career of the stigma concept has been shaped by various forms of sociological amnesia, misunderstanding, and misapplication. We point readers to Müller's^2^ overview of treatments of the stigma concept ‘after Goffman’ and find ourselves very much aligned with his assessments; not least his insistence that one really needs to read the whole of *Stigma* to understand, properly, what Goffman is up to in those first few, possibly misleading, chapters. Goffman's entire sociology does *not* proceed from the point of individual social actors on the one hand and societal structures on the other.^4^ Contemporary writings on stigma critical of Goffman (e.g., Tyler^12^) trade in the same ‘logic of exteriority’^13^ as Giddens' earlier critique. In other words, in attempting to ‘repair’ a perceived gap between Goffman's social actor and the historical and structural orders that he was apparently ‘blind’ to, much of this work reproduces a false relation between ‘structure’ and ‘agents’; a relation not recognized either in Goffman's work or interactionist sociology more generally.^14^

Outside of such a treatment, ‘stigma’ becomes a catch‐all concept for relations between ‘normal’ and ‘deviant’ categories. As various critiques of contemporary usages have noted, it is enroled to gloss relations and practices of shame, degradation, ‘self‐stigma’ and other *interactional* phenomena worthy of careful treatment.^2^, ^6^, ^15^ Conceptual clarity is not quite our concern here. We are, instead, concerned with how stigma is invoked as an explanation without consideration of the *production* of stigma in actual situations. Stigma is treated by social scientists as a thing of the world and, at the same time, a conceptual device for finding order in the world (in the manner critiqued by Garfinkel^7^ and the ethnomethodologists). It can be found operating as an *elevator concept*,^16^ adding a conceptual lift to mundane observations. ‘Stigma’ is similarly used as a *placeholder* concept, marking a conceptual, analytic space without the obligation of filling said space with necessary detail. Stigma is also, we suggest, a *bidet* concept. This likely needs further explanation. Years ago, Paul heard someone ask what the function is of a nonexecutive director in a company. The reply to the question was ‘It's a bit like a bidet. It adds a touch of class, but nobody knows how to use it’.

All of these (metaphorical) usages have something in common in allowing an author to do several things. A primary use is an affiliation of their work to a very broad range of sources; it offers professional membership in linking the work to some canonical texts to which readers can knowingly nod. Another is the deployment of ‘stigma’ in providing for a ‘black‐boxing’ of key analytic elements of their argument, in the very course of their objectivation.^17^ The practices which produce the context in and through which an account of ‘stigma’ is meaningfully produced are obscured by the very terms which serve to describe it. Such is glosses' work.

For reasons of brevity, we can only summarize general trends in recent stigma literature. They can be organized through the following observations:

1) Stigma is treated as a ‘thing’, attached to and sometimes even *equivalent* to a person. This means that the emphasis on *specific social settings* is lost^18^
2) Stigma is approached as a directly describable subjective ‘experience’.
3) The recognition that categories are *situated* and *always occasioned* is ignored in an insistence that attribute X *is* a stigmatizing predicate of social category Y.
4) Stigma is given agency, and is seen to ‘play a role’, or to be ‘promoting a view’.
5) Stigma is treated as a related to a permanent ‘label’ with structural causes *and* consequences in terms of, for example, access to resources and opportunities.
6) Many studies are based on interview data. That is not a problem per se, but it is not at all clear in such studies how authors are conceiving of that talk in relation to interactional phenomena, and actual social settings, in which stigma is accomplished (we try to demonstrate one way in which this might be done below).
7) Stigma is ‘bonded’ to other units of analysis and phenomena in a way that renders stigma as a ‘catch all’ concept, representing the kind conceptual vagueness that Goffman strenuously argued against across *all* his work.

A common feature of these uses is that ‘what everyone knows about stigma’ is made a resource, rather than a topic of inquiry in the construction of sociological commentary. In this way, although dressed in sociological garb, the use of term *precedes* real sociological endeavour (a common social scientific trouble produced in no small way by the formal processes of ‘research design’, ‘sampling’ and so on; analytic troubles are decided ahead of the field). Evidence for this argument includes Manzo's^18^ observation that ‘The social scientific ‘community’, such as it is, produces a delimited number of topics construable as stigmas’. We do not, for example, have a sociology of the stigma of being middle class. We must recognize that there are contexts in which Goffman's ‘unblushing’ American—if, indeed, they existed—could be stigmatized. Presumed ‘stigmatized’ categories trump the analysis of the production of stigma in any actual case. The *relations* that Goffman outlines in his treatment of stigma are substituted for writings about identities assumed to be problematic in one way or another by the analyst, quite ahead of any work that demonstrates *how* and in what ways, and what aspects of that identity are treated as problematic in which contexts. Relations of category, knowledge and identity are overlooked. Indeed, social scientists routinely put the cart before the horse, and proceed to drive it in a circle, by selecting a group that is understood to be stigmatized and proceeding to describe the experience of that group (usually elicited in interviews) as the experiences of not only *that* stigmatized group, but ‘the stigmatized’ as a whole. This potentiality was recognized by Goffman^1^ ^(p140)^ in his critique of the term ‘deviant’, noting that:

…those of us who live around the social sciences have so quickly become comfortable in using the term ‘deviant’, as if those to whom the term is applied have enough in common so that significant can be said about them as a whole. Just as there are iatrogenic disorders caused by the work that physicians do (which gives them more work to do), so there are categories of persons who are created by students of society, and then studied by them.

This position in relation to the analytic category ‘deviant’ reminds us that Goffman was attempting to shift the attention from the study of ‘deviants’ to an understanding of situated rule‐breaking which, again, is grounded in an understanding of the rules, demands and obligations that hold in each social setting.^19^

### 1.2 Beyond elevators, placeholders, and bidets

Admittedly, Goffman's own treatment of ‘stigma’ is not perfectly consistent. Nor is it unproblematic. It should, however, be remembered that attributes glossed as discrediting or discreditable can only be understood in terms of the judgements and actions of others, in their perceived and anticipated *evaluations* and *expectations*. Such evaluations and expectations are organized in relation to local treatments of categories, which are themselves *occasioned* by the context of their use.

In aiming to develop a properly situational sociology, Goffman's work on interaction order continually emphasized how context and the ‘definition’ of a situation gives rise to the actor's sense of personhood and standing. Some stigmata might be well be understood ‘to travel’ with the person, but the social significance of those stigma *must* be understood as produced, revealed, contested, managed, ignored and so on, in actual social settings. There are no immutable, a priori, stigmatic attributes of a person, precisely because, as Goffman highlights in the very first pages of the book, stigma is the product of social *relations*, not simply a matter of individual attributes. A situational approach would, thus, explicate *just how* and *just when* social relations—what we argue are *categorial* relations—give rise to stigma and the sorts of interactional troubles that follow.

In working out the properly situational approach outlined by Goffman, we think that some of the insights of Harvey Sacks, ^20^ and particularly as they were developed into what became ‘membership categorization analysis'^8^, ^9^, ^11^, ^21^, ^22^ are useful here. Drawing, albeit sparingly here, from this tradition, a first point is that—contra to most social science treatments—people do not straightforwardly ‘belong to’ this or that category, but, in a far more radical sense, the availability of a *relevant* category (and category device) organizes and accomplishes a person as a member of category, within a specific social setting and context. A second point is that any member can be categorized by an almost endless inventory of categories. That this is the case appears to be trivially obvious, and yet the question of *how* person‐description categories are produced and selected and made relevant in any case is a deeply analytic one for members and sociologists alike. As noted by Coulter,^23^ the analytic task is to demonstrate how a category becomes ‘operationally relevant’ in some scene, rather than rely upon its apparent perceptual assignability. A third is an alternate treatment of culture (which can be said to ‘produce’ the norms, values and rules which in turn create ‘normals’, ‘deviants’ and ‘stigma’), as *culture‐in‐action*.^8^ A situational treatment of stigma turns on a proper treatment of how categorizations are *actually* organized in any given setting. As suggested above, the stigma concept provides for the glossing of this categorial work routinely engaged in by members and social scientific analysts alike.

When it is said, for example, that ‘he might have got some help, if only there wasn't so much stigma around talking about mental health’, we can readily hear that there is a relation between the category ‘male’, the activity ‘seeking help for mental health’, and the assumed perception of *that* category of person engaging in *that* activity, in the eyes of some community (presumably ‘other men’, in this case). Thus, practical reasoning regarding social relations is necessarily categorial in character. Goffman,^1^ ^(p10‐11)^ of course, recognized this process:

Society establishes the means of categorizing persons and the complement of attributes felt to be ordinary and natural for members of each of these categories. Social settings establish the categories of persons likely to be encountered there. The routines of social intercourse in established settings allow us to deal with anticipated others without special attention or thought.

Note the relationship between context and category relevancy. What we need, then, are studies which describe *how* stigma is *occasioned* in and through and in local reference to categorial relations which are themselves radically local, endogenous, and unavoidably occasioned elements of any given scene.

It seems to us that developing Goffman's work in this way leads us to at least two possibilities for studies of ‘stigma‐in‐action’. The first would be the examination of stigma as *actually produced* in actual social settings. Such studies aim to describe *how* it is that stigma is accomplished in interaction, rather than constructing a social attribute (which is, usually, a social identity of some sort) as stigmatic at all points. The second possibility, which we illustrate in the remainder of this article, develops an attention to how, and in just what context, to do just what work, the gloss of ‘stigma’ is deployed by writers and speakers of both lay and professional sociology. We can begin with a brief description of some examples of the social scientific use of the concept in existing analyses of elicited talk where participants discuss the stigmatic aspects of mental health, self‐harm, in the context of experiences of seeking and receiving professional help.

### 1.3 The uses of the stigma concept in research on self‐harm

There is, in the research on self‐harm and suicide, an established discourse concerning the stigma of seeking helping and the shame associated with it. Alongside this, is the consideration of the negative experiences of help‐seeking and how this might discourage future efforts to explore support options. As a general orientation, this literature is characterized by the repeated treatment of negative experiences of seeking help and of interactions with care and support providing services as evidence of ‘stigma’ in clinical interactions. By and large, the interactions that produce the reported experience of stigma are not analysed and, by and large, relationships between an individual's reported experience and notions of stigma are done in an analysis which re‐renders the talk as talk about stigma of social scientific value.

In this literature, there is a recurrent running together of matters of losing face, of orientation to the perception of others, of shame, and of category membership and its positive recognition. An article by Mitten et al.^24^ provides an example of this tendency. In introducing the article, the authors advise that ‘stigma’ is deployed as an ‘overarching term’ and, indeed, it is used as such throughout the analysis and discussion. In the abstract they write, ‘The purpose of this study was to explore youths' perceptions of stigma…Results indicated that youth reported experiences of stigma from both clinicians and other patients, and some of these youth reported stigmatizing others with mental health disorders’. Here, we see a clear example of stigma treated as a ‘thing’ or, rather, in this instance, something that is ‘done’ or passed to a particular group (‘youth’) by others [‘clinicians’ and ‘other patients’ (heard as ‘not young’)]. ‘Stigma’ is constructed in and through its use as a *sociological* object—measurable and accountable, although not necessarily clearly defined—through accepted sociological means.

Youth were able to identify many experiences of stigma in their own lives, both that they had observed, and that they had experienced themselves. Youth (*n* = 10) shared the following as common stigmatizing reactions as invalidating when people learn about their mental illness/self‐harm:

Well, I don't know, kinda made me feel like my problems weren't like, valid, I guess because they were there for I don't know, just made me feel like my problems weren't valid. … Um, really its just people being like, well you don't look sick and me being like it's a mental disorder, you don't look like you have cancer, but you do. Like, you can't say that, you know ‘(Participant 1)’.

We can note how what 10 people report is treated as representative of the category ‘youth’, which is, here, related to the activity of identifying experiences of stigma. The issue is strictly *not* a matter of extrapolation from a sample—much can be done with a sample of *n*= 1—but, rather, what this talk is made to do in and through the introduction of the concept of stigma. The account itself deals with a matter of the perception of others in relation to the individual's ‘mental disorder’. There is a contrast constructed between the ‘internal’ condition and the externally available appearances. The account is underpinned by categorial logics in making sense of the experience of Participant 1 (who now stands as a representative member of the category ‘youth’ and ‘sufferers of a mental disorder’) and their treatment by another group: ‘people’ (which is hearable as non‐members of either category). These interactions are accounted for as the breaching of some rule, or the inability to meet certain demands and expectations, for which the category ‘mentally ill’ might have some mitigating or explanatory effect. This kind of categorial trouble is not necessarily equivalent to stigma, and yet is made to stand in for it in the methodologically ironic social scientific ‘correction’ of mundane practical reasoning. This, again, establishes the ‘normal/stigmatized’ two‐set category pair^20^ which is present in the account *and* the analysis by the authors. Stigma as a concept is deployed ahead of the talk, to make a formal analytic matter of the relatively mundane, selected, remarks of the participant which, in our reading at least, have more to do with the speaker's reported troubles in making relevant the category ‘mentally ill’ in unspecified interactions with others.

To repeat the main point of the article, if we are to take social relations describable as ‘stigmatic’ seriously, then we must recognize that rolling out stigma as an elevator, place‐holder, or bidet concept obscures the actual lived detail that produces both the affective experience of a ‘stigmatic’ encounter, as well as related practices of degradation, discrimination and devaluation. The stigma concept, deployed as a catch‐all, simplified construct, can obscure the lived detail of the complex social relations that shape care‐experienced young people's encounters.

In the same paper,^24^ ^(p9)^ another participant produces an account grounded in this same categorial reasoning, which closely matches the sociological use of the concept:

One youth explained her understanding of stigma, and described how people with mental health are divided and labelled:

The overall picture is stigma can really separate you from the rest of the population. In terms of you're a crazy person, and these are normal people. And like, it's not something that, that type of stigma is a little more discrete. It's kind of, nobody will say that to you, outright, it's just how you are treated, and there's no faith in you. (Participant 5)

Here, we can note that the stigma concept can be deployed in the construction of contrasts that invite interactants to find relevant differences, and to make consequential moral evaluations. Contrastive devices are characteristic of everyday and professional talk. Smith^25^ for instance analyses a description of the behaviour of another (‘K’) that invites the hearer to find particular instances as evidence for eccentricity or even mental illness. Contrastive talk is also used by professionals to make implicit, or even explicit, comparisons between their own practice and the work of others (found to be wanting in some way). Atkinson^26^ documents such contrastive talk on the part of American haematologists, who distinguished their own diagnoses with the apparent shortcomings of physicians ‘elsewhere’. Stigma has been explicitly addressed in this vein by discursive psychologists.^27^ They argue, building like us on Garfinkel and Goffman, that the analytic resources of discursive analysis need to be brought to bear on ‘stigma‐in‐practice’. Attention to the discursive work of invoking and attributing ‘stigma’ may be some distance removed from Goffman's original conception of spoiled identity, but attention to how stigma is used as a discursive resource remains faithful to the general spirit of the sociological and social‐psychological origins. It is an approach that still resists the dilution of stigma as an analytic concept.

In the follow section, we point to an alternative approach to analysis of ‘stigma talk’ and highlight the shared modes of (categorial) reasoning employed in social scientific and lay accounts.

### 1.4 Talking stigma: members' constructions and uses of a concept

In further pursuing the categorial logics that underpin the use of the stigma concept, the following data excerpts are taken from a study of foster and residential carers' experiences of preventing and managing self‐harm among care‐experienced young people, conducted between 2015 and 2018.^28^, ^29^ The excerpts below are drawn from a focus group in which in which the mental health and wellbeing of young people with experience of being in care is discussed. Rather than treating such accounts as providing direct and measurable access to stigma as an object, we aim to demonstrate how ‘stigma’ is presented as a resource for mundane and practical reasoning relating to difference and deviance and, in this way, how ‘stigma’ is inter‐subjectively understood and ‘objectified’ within the talk, as an accounting device providing for generalized cultural expectations.

*Data Except 1*

1. R1: And again, it comes back to this looked‐after child. He's different
2. or she's different because they are looked after and some of the
3. children see them that way. And they put pressure on them. But
4. then it's up to the teachers when they are in school to protect those
5. children as well and they are not doing that, are they, at the
6. moment.
7. R2: But there's a massive stigma with her because ‘like if I wasn't looked
8. after I could do this. Why are you stopping me?’ Because I try to
9. explain, it doesn't matter what you are going to be doing, you are
10. always going to have that wherever you go. Whether it's in school,
11. whether it's here, there or everywhere, you are always going to have a
12. label of a looked after child. And you are different. And that is an
13. issue with some of them.

This excerpt features talk between two carers. The accounts sense‐making operates in relation to perceptions, actions, and obligations of others (fellow pupils and teachers) and their own work with and through the stigma concept. The construction in lines 1−3 is of a generalized ‘looked‐after child’, rather than a specific individual, and goes on to build a similarly generalized partitioning of those children, by virtue of that attribute, from others. The product of that work, being here the construction of a complaint about teachers' lack of protective action for this population, within that setting in order to produce a complaint about teacher's responsibilities (l. 4−6). This account is then elaborated by R2 with a contrastive account which switches to their troubles with working with the attribute ‘looked after’. Here concept ‘stigma’ is introduced as a precondition for the experiences of the child, which is then explained through the differential status of the child and, reportedly, the carer's own protective actions and treatments of participation in activities and settings. Something of this work relating to the attribute ‘looked‐after’ is observable in the next excerpt in which the talk concerns the expected, and again generalized, perception of actions done by members. The reasoning thus turns on a members' treatment of the categorization of actions in relation to the doer in relation to the treatment of behaviours and applicability of the action‐categorization ‘self‐harm’.

*Data Except 2*

14. I: Do you think there is an increased tendency to interpret ‘normal’
15. behaviours, so to speak, as self‐harm, just because they are looked
16. after?
17. R: Umm, people do have a negative view of looked after children. I
18. personally have not had this said to me but I have friends that have it
19. said to them, ‘oh so you look after naughty children?’, And umm, it
20. ((laughs))
21. the sort of stigma is there and this child is out of the ordinary
22. and perhaps the survival technique we have embedded in us, if
23. something is not normal then we need to watch it
24. ((laughs))

Here, the interviewer works with the two‐set category pair ‘deviant/normal’ to put the idea on the table that ‘normal’ behaviours (i.e., actions that are ‘category free’ in that they could be done by anybody), are recategorized as ‘self‐harm’ in and through the availability of the category ‘looked after’. This suggestion is treated, with apparent caution (the ‘umm’ [l. 17]), with a revision which, again, first produces a relationship between the category and an attribute (of being naughty) that is ‘out there’ as thing that has conceivably being said, before moving on to invoke stigma as a *product* of that relationship with consequences of (again) a partition established between the ‘normal’ and the ‘deviant’.

Whilst we, of course, recognize that what we have been working with here is abstracted interview, we do suggest that the materials provide for an insight in the categorially‐based reasoning that underpins both lay and professional sociologies of stigma. This is, in the absence of access to clinical interactions themselves (described across the rest of this special issue), one way in which to avoid a key critique of the mainstream treatment of deviance and difference^30^; that, on close inspection, ‘labelling accounts’ reveal an overwhelming preoccupation with generalized descriptions of cultures of understanding, often elicited from interviews in the field, allegedly brought to bear upon interactional scenes.

## 2 CONCLUSION

In this article we have argued for an attention to the work that the stigma concept does in social scientific and lay discourse. We do so in order to demonstrate that ‘stigma’ is not a ‘thing’ in itself, but arises in social interaction and can be used as a device for making sense of problematic social relations. In this way, we have approached ‘stigma’ as a linguistic construct of *natural language* with a *technical* referent in the social sciences. We have returned, briefly, to some of Goffman's original formulations of stigma in order to develop a critique of some of the contemporary treatments of the concept. We have argued that formal analytic, theorized, version of stigma—that attempts to reconcile the position of the individual in relation to assumed social and historical structures—has misplaced its critique of Goffman, due to a number of abstractions from both Goffman's sociology and the observations of settings and interactions in which stigma (and degradation, loss of face, shaming, discrimination and so on) is accomplished in and through and as a ‘language of relations’. We then moved on to consider the uses of ‘stigma’ as a concept in both professional social scientific and lay sociological reasoning. In both analyses, we aimed to at least point to how ‘stigma’ is routinely deployed as a gloss with which members readily make sense of a whole range of matters of membership, belonging, exclusions, identity, shame, motivation, action and in action and so on. In what could only be an illustrative analysis, we have also pointed towards how this practical reasoning is categorially organized. We have not had room to fully elaborate this here but suggest that a necessary critical engagement with Goffman's treatment of stigma can be found in the ethnomethodological treatment of categorization practices. What we have hinted at, but not at all developed is how the discussion of the ‘stigmatized’ and ‘normals’, and case‐specific variants thereof, can be shown to be routinely embedded in both social scientific and lay analyses, accomplished through the recurrent construction of a two‐set category class. As Harvey Sacks observed^20^; ‘Two‐set classes would seem to have certain kinds of attractions… they're tremendously easy to compare… you can apparently make an observation of comparative lack much more easily than otherwise’. Sacks goes on to note that all sorts of social arrangements, from conflicts and revolutions, to games, are organized in terms of two‐set classes, the ‘haves’ and the ‘have nots’, and speculates that formulating social relations as a two‐set class appears to be ‘a method of doing things’ that might be significant as a device that forms ‘a basic mechanism of social control’. Whether and how that is the case requires further examination. Either way, the orientation to ‘stigma’ outlined here, finds the ‘machinery’ which *occasions* (discussions of) stigma, located not in social structure or exterior historical process but, rather, as existing in the mundane, diffuse and endogenous categorization practices employed in production of members' accounts of the organization of any given scene. That is a step towards working out the ‘language of relations’, which, in turn, does not rely on the analytic use of ‘stigma’ as a comforting and convenient conceptual gloss. In terms of the degree to which a social scientific analysis might contribute to improving encounters in clinical settings, this step may go a long way toward recovering what is submerged in standing treatments of ‘stigma’.

## ACKNOWLEDGEMENTS

This work was supported by The Centre for Development, Evaluation, Complexity and Implementation in Public Health Improvement (DECIPHer) funded by Welsh Government through Health and Care Research Wales. The author(s) disclosed receipt of the following financial support for the research, authorship, and/or publication of this article: This work was funded by the National Institute for Social Care and Health Research (NISCHR) (SCF‐14‐09) in Wales. The views expressed in this publication are those of the authors and not necessarily those of NISCHR.

## 3 CONFLICT OF INTEREST

The authors declare no conflict of interest.

### DATA AVAILABILITY STATEMENT

Research data are not shared.
